# Assessment of the dynamics of concentration and competitive positions of the Baltic cruise port system

**DOI:** 10.1186/s12544-022-00532-7

**Published:** 2022-04-01

**Authors:** Jerónimo Esteve-Pérez, José Enrique Gutiérrez-Romero

**Affiliations:** Department of Applied Physics and Naval Technology, Universidad Politécnica de Cartagena, Paseo Alfonso XIII, 52, 30203 Cartagena, Spain

**Keywords:** Cruise shipping, Baltic Sea, Port competition, Port competitive position, Dynamic portfolio analysis

## Abstract

**Objective:**

This study aims to investigate the degree of concentration and the competitive positions of the Baltic cruise port network.

**Methods:**

A set of 29 Baltic ports are analysed, market concentration is evaluated using the analytical technique of the Herfindahl–Hirschman index, and competitive positions are determined through portfolio analysis based on the Boston Consulting Group matrix from 2000 to 2019.

**Results:**

The Herfindahl–Hirschman index indicates that the Baltic cruise port system is unconcentrated with an average score of 0.11 for the analysed period, suggesting that eight of the twenty-nine ports are the dominant ports in the Baltic. Portfolio analysis results suggest that the hierarchy picture of competitive positions is dynamic and has changed over time. The Baltic cruise port system has a wide range of competitive positions. Kiel and Rostock becoming mature leaders is one of the most relevant changes in competitive positions.

**Implications of the research:**

This study contributes to the literature not only by investigating the competitive positions of the second most important European operational area for cruise ships but also by filling the gap in research on the concentration and competitive strategic positions of Baltic cruise ports. This research allows seaport operators to visualise the position and progress of selected ports and predict the possible future seaport developments.

## Introduction

Cruise traffic is registering a remarkable dynamism worldwide that implies a year by year increase in the number of people who choose to cruise as a holiday option. This positive trend has been negatively affected by the SARS-CoV-2 pandemic. The increase in demand for cruises necessitates the ordering of new ships by cruise lines. During the 2000–2019 period, the number of people worldwide who chose a cruise to enjoy their holidays grew 7.6% yearly, on average [1]. Moreover, during the same period, 167 cruise vessels were put into service, with an associated capacity of 385,000 onboard beds per day [2].

The cruise industry has a global capacity deployed primarily in Alaska, Asia, Australia/New Zealand/South Pacific, Caribbean Sea, Mediterranean Sea, Northern Europe, and South America. The Caribbean Sea is the leading destination region; for example, in 2019, it accounted for 34.2% of the deployed capacity. The cruise traffic in Europe is concentrated in the Mediterranean Sea and Northern Europe, and both destination regions accounted for 28.4% of the deployed capacity in 2019. Specifically, the Mediterranean accounted for 17.3% (second leading destination region worldwide), while Northern Europe accounted for 11.1% of deployed capacity [3]. The Northern Europe cruise destination region can be divided into four subregions: Atlantic Europe, Baltic Sea, United Kingdom and Ireland, and Norway, Iceland and Faroes Islands.

Compared with other popular cruise destinations, there is a lack of studies focusing on the Baltic Sea as an operational area for cruise ships. The studies on cruise traffic primarily focus on the two main destination regions worldwide, i.e., the Caribbean and the Mediterranean. In this regard, one can find the works of Hall and Braithwaite [4], Lester and Weeden [5] and Wood [6] focusing on the Caribbean Sea and the works of Esteve-Perez and Garcia-Sanchez [7], Lorenčič et al. [8], and Pallis and Arapi [9] focusing on the Mediterranean Sea.

Studies focusing on the concentration and consolidation of seaports in a region or a country are important to better understand how a port system develops and evolves in terms of the distribution of cargo among ports and the level of competition in a particular region or country [10]. For example, De Oliveira and Cariou [11] suggest that port efficiency can be compromised when greater interport competition occurs at the regional level. The organisation’s competitive position is defined in a study by Fleisher and Bensoussan [12] as the position of an organisation compared to its competitors in the same market or industry. Knowledge of this item allows enterprises to make tactical plans to maintain or improve their current positions or possibly withdraw from the market. Therefore, knowledge of this issue is critical.

In the case of cruise ports, it is interesting to know the competitive positions of the ports in a destination region because the cruise ports cooperate and compete simultaneously. A cruise port needs a set of surrounding ports to establish an itinerary, and then needs to create a demand for this itinerary. Finally, apart from the general competitive structure of the port network, ports also draw cooperative strategies [13]. This cooperation is mostly observed in adjacent ports, forming latent groups of ports within the cruise network.

Taking as reference (a) the global evolution of the cruise industry, (b) the strategies to design itineraries, (c) the features of the Baltic Sea as operational area for cruise ships, and (d) the lack of studies about cruise shipping in the Baltic Sea, some questions arise about the dynamics of cruise shipping in the Baltic Sea that are addressed in this study: Which are the dominant ports in the Baltic cruise port network?; How concentrated has the Baltic cruise port network become?; and How has the ‘hierarchy’ of competitive positions changed among the Baltic cruise ports?

Therefore, the purposes of this study are to determine the concentration of cruise ports in the Baltic Sea, investigate their competitive positions and suggest recommendations on cruise port operation based on the obtained results. To this end, the market concentration among 29 Baltic cruise ports is evaluated using the measure Herfindahl–Hirschman index (HHI). In addition, the competitive positions of the top 15 major cruise ports in the Baltic Sea are determined through dynamic portfolio analysis.

The remainder of the paper is structured as follows. Section 2 provides a review of the literature to contextualise the empirical study, presenting the major issues of cruise shipping and the features of the Baltic Sea as an operational area for cruise ships. Section 3 presents the methodology to calculate market concentration and to determine the competitive positions of the Baltic cruise ports. Section 4 shows the results of the market concentration analysis and the dynamic portfolio analysis. In Sect﻿. 5, the implications of the results are discussed in light of (1) a comparison of the findings with previous research, (2) theoretical and practical implications of the research, and (3) limitations of the research. Section 6 ends the study with the conclusions and future perspectives of the research.

## Literature review

### Cruise shipping

Cruise traffic is a complex shipping business because the cruise vessel has the threefold function of accommodation facility, means of transport and tourist attraction. During the 2010 decade, the number of scientific works devoted to the cruise industry increased compared to the scientific works published in the two previous decades. Studies have investigated topics such as customer loyalty [14], the sustainability of the cruise industry [15, 16], seasonality of cruise operations [17–19], cruise passengers’ satisfaction at destinations [20], cruise supply chain and its disruptions due to natural phenomena [21], home-porting selection criteria [22], residents’ perceptions of cruise tourism [23], and challenges of the cruise industry [24].

Focusing on the design of cruise itineraries, they are executed via the deployment of vessels in a specific geographic cruise region [25]. The cruise product has become diversified to attract new customers and to respond to the wide array of customer groups [26]. From a geographical point of view, a cruise itinerary encompasses three areas: (1) the sea area where the itinerary occurs; (2) the ports, homeport and ports of call that compose the itinerary; and (3) the tourist hinterland visited in each call.

According to Wang et al. [27], the distance of ports included in an itinerary affects the overall schedule of cruise companies due to cost and time efficiency issues. Moreover, ports’ proximities to major tourist attractions have a notable effect on passengers’ preferences among different offered itineraries [28]. At the itinerary planning stage, cruise companies have to select among a set of ports encompassing various geographical attributes to build attractive itineraries for passengers and thus fulfil their primary target of profit maximization [29].

Cruise lines are continuously applying strategies to expand the cruising activity and thus increase the potential number of people becoming cruise passengers. There are the main strategies developed by the cruise lines, which are linked with itinerary redesign [25, 26]. The first is the exploitation of economies of scale and scope, with larger cruise vessels hosting more passengers, lowering operating costs per passenger. Moreover, through this strategy, cruise lines provide enriched, upgraded, and differentiated on-board amenities, facilities and services that allow on-board market segmentation [22]. The leading cruise companies have ambitious and highly capital-intensive investment plans under development, with an active newbuilding order book for vessels of larger carrying capacity, expensive technological advances, and modern facilities to cater to diversified cruise passenger needs, complying at the same time with strict environmental conditions [30]. The second is the endorsement of deployment strategies eying the expansion of the number of destinations included in cruising itineraries, calling new and most popular markets at an extend that regularly tests the carrying capacity of port-cities and destinations [22, 31]. The third element is their expanding presence in cruise terminal operations and port governance [32].

Regarding the redesign of itineraries and strategies of the cruise lines to attract the attention of cruise passengers, it is of interest to address the features of the Baltic Sea as a cruise destination region. It is also of interest to address the performance of its ports in terms of concentration and competition to fill the research gap in the Baltic Sea. Through this research, the authors contribute a set of challenging and innovative findings about the dynamics of the Baltic Sea in terms of the concentration and competitive positions of its ports.

### The Baltic Sea as an operational area for cruise ships

The Baltic is a closed sea that is connected with the Kattegat and Skagerrak straits in the North Sea through the three Danish straits. One major reason for the Baltic Sea being an attractive cruise destination is that it is the sole region in Northern Europe with six capital cities situated on the coast and within overnight sailing distances of each other [33]. Additionally, the Baltic region is not only home to some important European capitals but also has many small hidden gems that offer a relaxing environment close to nature [34]. This feature is crucial because for a region to be successful in cruises, it needs to configure itineraries that combine ports with different types of available tourist attractions. Specifically, it is necessary to combine marquee ports with other types of less popular ports. Furthermore, there should be a balance between sailing time and port time.

Lundgren [35] stressed some advantages of the Baltic Sea, such as its sheltered location, easy navigation and land-based transport infrastructure improvements. Charlier and McCalla [36] highlighted the Baltic Sea cruise market’s seasonality. Because of the climate, the market potential in the region is short compared to other cruising destinations. The season stretches from April to September, with a peak in mid-summer. In the wintertime, there is hardly any offer of cruising at all in the Baltic Sea area.

The Baltic Sea has rough conditions for sailing during winter due to icing. The maximum ice thickness during winter varies depending on the concerned area of the Baltic Sea. Ice thickness can be divided into fast ice (fastened to the shore and unable to be pushed away by the wind) and sea ice found off the shore. On the one hand, the hardest ice conditions are registered on Northern Bothnian Bay, with a mean sea ice thickness of 44 cm and an extreme value of 70 cm. On the other hand, the lowest ice conditions are registered in the Archipelago Sea, with a sea ice thickness of 15 cm and an extreme value of 25 cm [37]. The above ice conditions generate navigation restrictions during some periods of the year. For instance, Bothnian Bay registered the most extended restriction period, from January to April. Vessels with minimum ice-class notation are required to sail in the whole Baltic Sea in winter [38]. However, new polar class cruise vessels could open new opportunities.

**Fig. 1:**
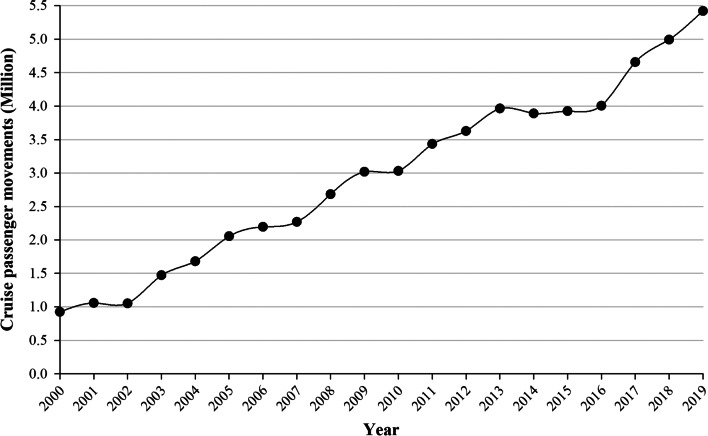
Evolution of cruise passenger movements in the Baltic Sea during the period from 2000 to 2019. *Source*: Author’s elaboration based on data from Cruise Baltic [39]

A 10% annual average growth of cruise passenger movements was registered in the ports of the Baltic Sea during the 2000 to 2019 period. Since 2013, the milestone of 4 million cruise passenger movements in the set of Baltic ports has been exceeded (see Fig. 1).

The number of ports hosting cruise ships increased in the same period. In 2019, 29 ports registered cruise calls, while in 2000, the number of ports hosting cruise ships was 21 (see Table 1). This trend is a consequence of the growing interest of ports in hosting cruise ships and the cruise lines’ need to include new ports in their itineraries. Specifically, the cruise lines need to vary the attractions offered to cruise passengers.

**Table 1:** Cruise passenger movements of 29 Baltic ports in 2000 and 2019 and cumulative and average cruise passenger movements for the period 2000–2019. *Source*: Author’s elaboration based on data from Cruise Baltic [39]

Port	Cruise passengers in 2000	Cruise passengers in 2019	Cumulative cruise passenger movements	Average cruise passenger movements per year
Copenhagen	166,000	940,000	11,711,500	585,575
St. Petersburg	149,252	666,235	7,951,840	397,592
Stockholm	157,000	656,443	7,702,253	385,113
Tallinn	109,511	656,000	7,558,139	377,907
Helsinki	140,000	603,500	6,643,000	332,150
Kiel	48,033	803,000	6,002,693	300,135
Rostock	52,622	634,000	5,971,769	298,588
Oslo	108,813	229,068	4,042,597	202,130
Visby	48,339	100,956	1,233,232	61,662
Riga	–	69,207	1,202,118	63,269
Gothenburg	3400	108,800	837,800	41,890
Klaipeda	4613	68,129	709,023	35,451
Aarhus	12,868	58,884	695,551	34,778
Rønne	12,000	19,692	342,087	17,104
Gdansk	3643	22,411	238,524	11,926
Skagen	142	66,258	233,312	11,666
Aalborg	400	40,223	161,129	9478
Helsingborg	6266	3812	155,455	7773
Malmö	–	28,800	141,490	10,884
Mariehamn	1678	13,304	105,646	5282
Kalundborg	–	1803	93,976	7229
Turku	5654	1364	88,618	4664
Saaremaa	–	28,336	69,060	4933
Fredericia	–	19,735	62,882	10,480
Kalmar	4100	655	53,332	2807
Luebeck‐Travem	–	5600	47,869	11,967
Elsinore	400	1172	39,603	2200
Karlskrona	–	600	39,223	2179
Kotka	–	7513	19,837	3306

Among the 29 ports that registered cruise passenger movements in 2019, five exceeded 100,000 passenger movements per year since 2000. These are Copenhagen, Helsinki, St. Petersburg, Stockholm, and Tallinn. Copenhagen is the leading port, with a throughput of 11.7 million cruise passenger movements between 2000 and 2019 (see Table 1).

The set of Baltic ports has a mix of homeports, ports of call and part-homeport. In homeports, the start and end of the itinerary occur. In ports of call, a cruise ship remains for a limited number of hours; during this time, cruise passengers will visit the port’s tourist hinterland [18]. A part-homeport is defined as a port in which at least 25%, but not all passengers, leave the ship and new passengers board [40]. The role of each Baltic port is shown in Table 2.

**Table 2:** Roles of Baltic ports analysed. *Source*: Author’s elaboration based on data from Cruise Baltic [39]

Type of port	Ports
Homeport (also port of call)	Copenhagen, Kiel, Rostock, Stockholm, and Helsinki
Part-homeport (also port of call)	Gdansk, Gothenburg, Luebeck, Malmö, St. Petersburg, and Tallin
Port of call	Riga, Visby, Klaipeda, Aarhus, Rønne, Helsinborg, Skagen, Aalborg, Kalundborg, Turku, Mariehamn, Kalmar, Saaremaa, Karlskrona, Elsinore, Fredericia, Luebeck, and Kotka

**Fig. 2:**
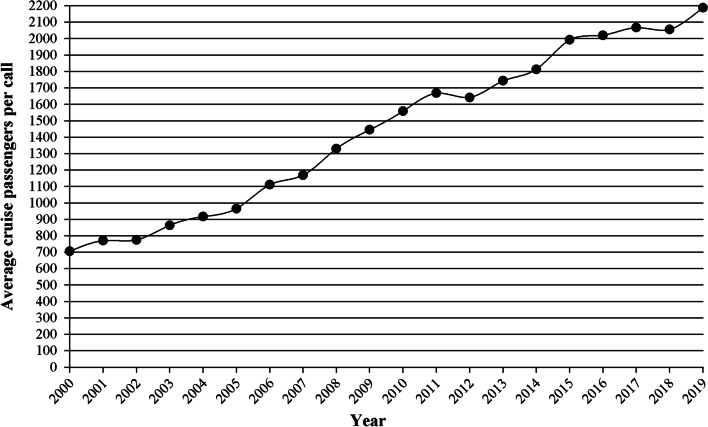
Evolution of cruise passenger movements per call in the Baltic ports during the period 2000–2019. *Source*: Author’s elaboration based on data from Cruise Baltic [39]

The Baltic ports have also witnessed an increase in the capacity of the new vessels put into service. The average cruise passengers per call in Baltic ports changed from 705 in 2000 to 2187 in 2019 (see Fig. 2). This means an average yearly growth of 6.2% during the period from 2000 to 2019. Therefore, Baltic ports have faced the contemporary challenge of cruise ship gigantism. Additional opportunities for onboard sources of revenue have been created through the application of economies of scale, yielding to mega-cruise ships with vast passenger capacity. New ship designs include new entertainment concepts, facilities and services.

## Research design and methods

**Fig. 3:**
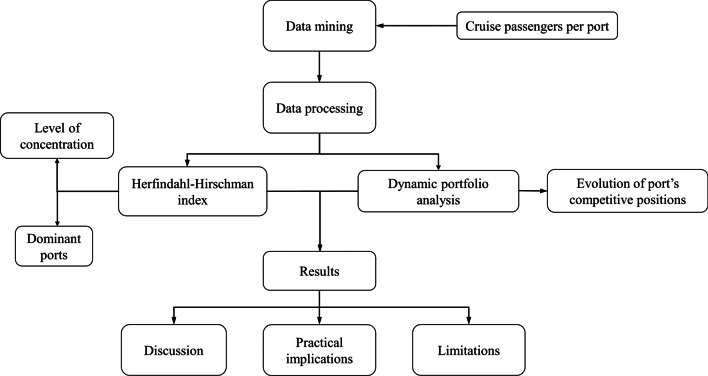
Research design flow chart. *Source*: Author’s elaboration

The research design is summarised in Fig. 3. In the data mining step, the information to conduct the analysis was retrieved. In data processing, techniques to calculate concentration and to identify the competitive positions in the Baltic cruise port network were applied. Both research steps will be explained.

Regarding data mining, the total cruise passenger movements registered in each port was the variable selected to perform the analysis. This variable was selected because it has the highest precision in measuring the cruise traffic throughput registered in each port. The total cruise passenger throughputs of 29 Baltic ports were analysed in the 2000–2019 period. The source of the data was the passenger statistics report of Cruise Baltic [39]. Ports with more than 10,000 cruise passenger movements per year from 2000 to 2019 were selected for the dynamic portfolio analysis. A set of 15 ports fulfilled the above criteria. The studied period was divided into the subperiods of 2000–2004, 2005–2009, 2010–2014 and 2015–2019 to assess the positional variations of the selected Baltic ports over the time period.

The first stage of data processing is to calculate the level of concentration in the cruise port network as a proxy to measure competition in the Baltic cruise port market. According to Zhang et al. [41], Yuen and Zhang [42], and Yuen et al. [43], the HHI can be used as a proxy to determine the level of market competition since the competition is inversely related to the concentration [44]. For instance, the research carried out in the works of De Oliveira and Cariou [11], Pallis and Arapi [9], and Varan and Cerit [45] used HHI as a proxy for inter-port competition among different players in a market. Other studies [7, 46, 47] also used the same approximation for their research. The HHI measures the degree of concentration in the cruise port industry and is calculated as follows Eq. (1):

1 $${\text{HHI}} = \mathop \sum \limits_{i = 1}^{n} \left( {\frac{{Cruise \;Pax_{i} }}{{\mathop \sum \nolimits_{i = 1}^{n} Cruise\; Pax_{i} }}} \right)^{2}$$

where *HHI* is the concentration index for the cruise port sample, *Cruise Pax*_*i*_ represents the cruise passenger throughput of the i﻿th cruise port and *n* is the number of ports in the port sample. The HHI ranges from 1/n to 1. An HHI value of 1 indicates total concentration in the industry, showing that the market is dominated by one specific cruise port (monopoly). On the other hand, the industry is perfectly competitive (pure competition) if the index reaches the minimum value of 1/n, where the market share is divided equally for all cruise ports.

Increases in the HHI generally indicate a decrease in competition and an increase in market power. An HHI index below 0.01 indicates a highly competitive index, below 0.15 indicates an unconcentrated index, between 0.15 and 0.25 indicates a moderate concentration, and above 0.25 indicates a high concentration [9]. Therefore, its inverse, *n*_*e*_ = *1/HHI*, describes an equivalent cruise market with *n*_*e*_ ports having the same market share. Consequently, *n*_*e*_ is interpreted as the number of dominant ports in the market [8].

The second stage of data processing was to identify the competitive positions and their evolution in the cruise port network. A portfolio analysis based on the Boston Consulting Group (BCG) matrix was performed to determine how the competitive positions of the Baltic ports have changed over time. A dynamic portfolio analysis aims to show the evolution of seaports within certain temporal frameworks and for given types of cargo to help port stakeholders draw conclusions or predict the future development possibilities of seaports [48]. Therefore, through this approach, we can obtain more accurate results because portfolio analysis provides a dynamic view of the progress of port positions over a distinct period [49]. In this case, the version of the BCG matrix adapted to the port industry has been used. The horizontal and vertical axes represent the relative market shares and annual average growth rates, respectively. The matrix is divided into four distinct sections: ‘High Potential’, ‘Star Performer’, ‘Mature Leader’ and ‘Minor Performer’. ‘High Potential’ indicates a port with a high growth rate and a low relative market share. The ‘Star Performer’ position is occupied by ports with both a high growth rate and market share. ‘Mature Leader’ is represented by ports with a high market share but low growth rate. The ‘Minor Performer’ position indicates both a low level of growth rate and a low market share.

## Results

**Fig. 4:**
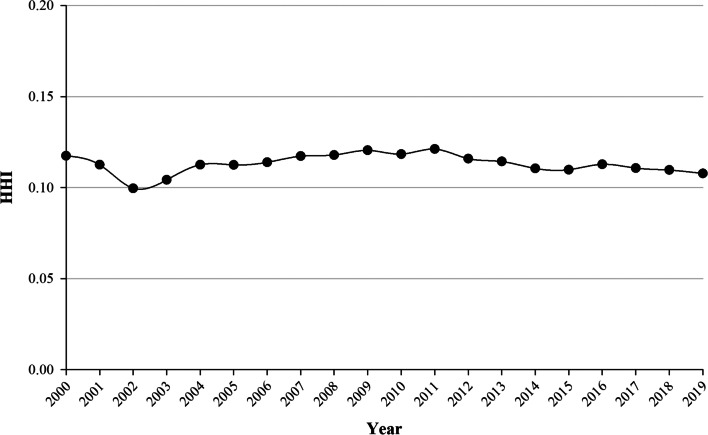
Evolution of HHI from 2000 to 2019 for the set of Baltic ports analysed

Figure 4 shows the change in market concentration in Baltic cruise ports described by HHI. The HHI is steady and relatively low over the two decades analysed, and the HHI ranged from 0.1175 in 2000 to 0.1078 in 2019. This fact implies that cruise traffic is unconcentrated. This shows that the cruise market in the Baltic Sea is dominated by approximately 8 ports (out of 29 ports): Copenhagen, St. Petersburg, Stockholm, Tallinn, Helsinki, Rostock, Kiel, and Oslo.

**Fig. 5:**
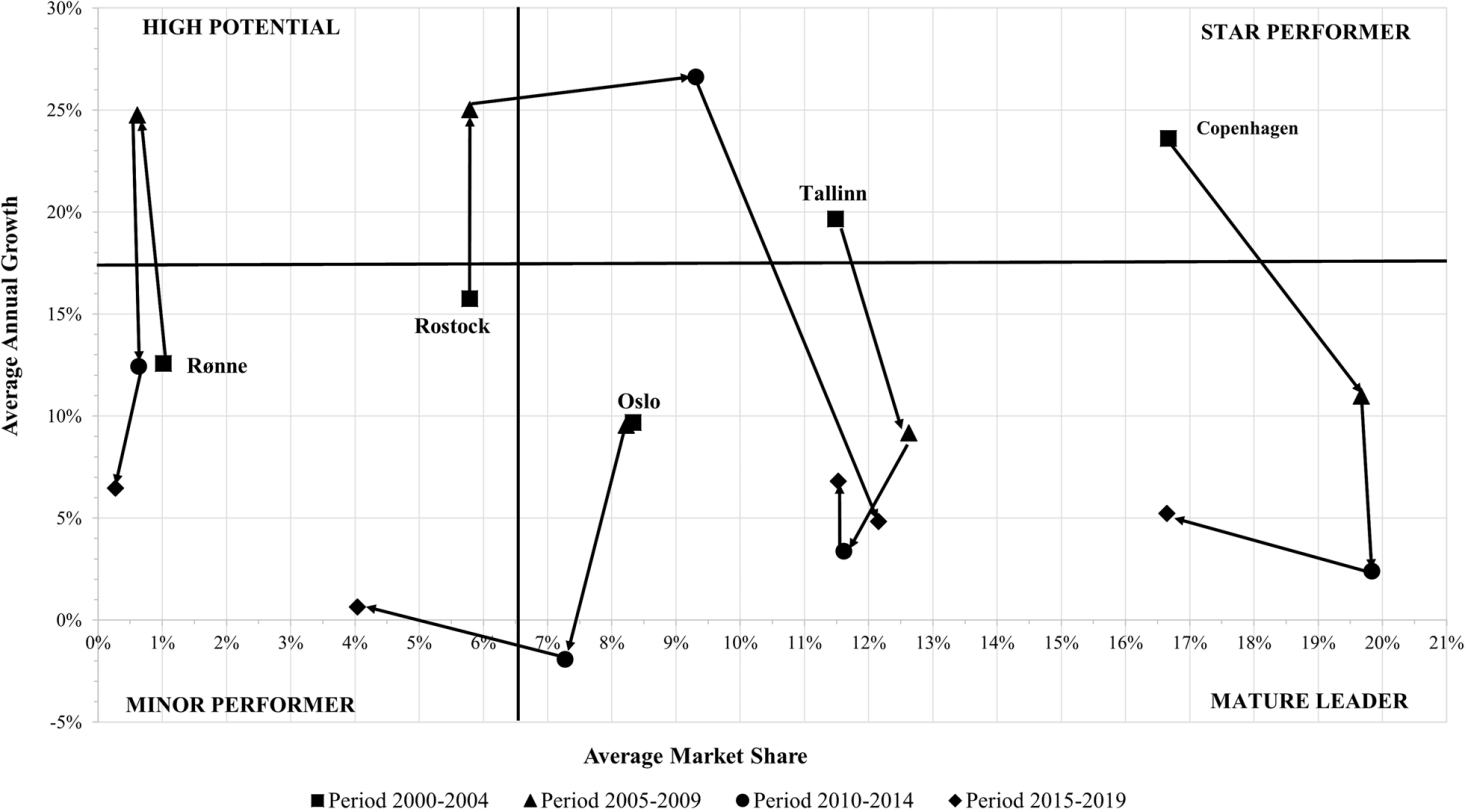
Dynamic portfolio analysis of ports of Copenhagen, Oslo, Rønne, Rostock and Tallinn during the 2000–2019 period. *Source*: Author’s elaboration

**Fig. 6:**
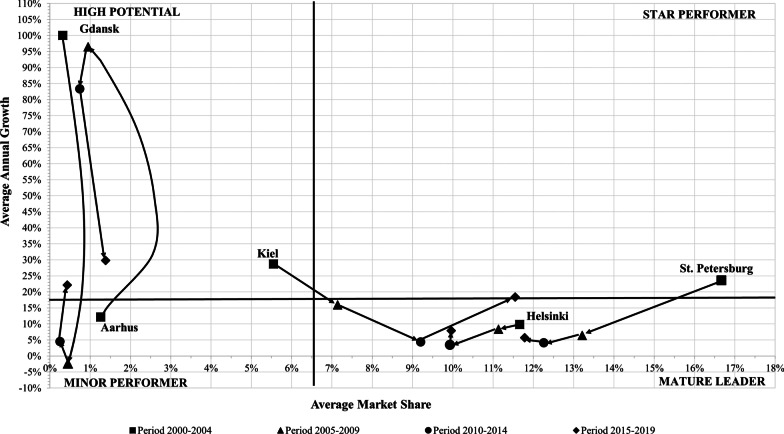
Dynamic portfolio analysis of ports of Aarhus, Gdansk, Helsinki, Kiel and St. Petersburg during the 2000–2019 period. *Source*: Author’s elaboration

**Fig. 7:**
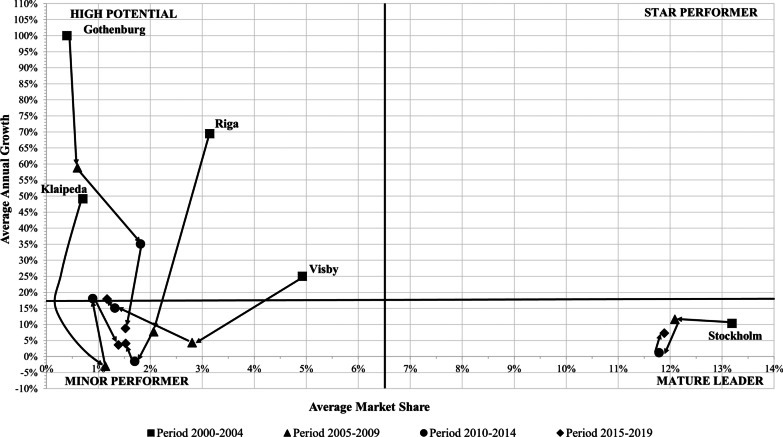
Dynamic portfolio analysis of ports of Gothenburg, Klaipeda, Riga, Stockholm and Visby during the 2000–2019 period. *Source*: Author’s elaboration

Figures 5, 6 and 7 show the results of the dynamic portfolio analysis. The ports of Copenhagen, St. Petersburg and Tallin showed competitive improvement. They moved from a ‘Star Performer’ position in 2000–2004 to a ‘Mature Leader’ position in the following years (see Figs. 5, 6). The German ports of Kiel and Rostock had competitive improvement. Rostock was positioned in a different quadrant in each subperiod. It evolved from the ‘Minor Performer’ position in the 2000–2004 subperiod to the ‘Mature Leader’ position in the 2014–2019 subperiod (see Fig. 5). Kiel started with the position of ‘High Potential’ and reached the ‘Star Performer’ position during the 2014–2019 period (see Fig. 6). The competitive improvement of Rostock and Kiel shows a remarkable increase in the market share of both ports. This growth is related to the character of home ports for the German source market, which has been the main European source market since 2015, reaching 2.59 million cruise passengers in 2019 [50]. That is, these ports are the closest gateways to the Baltic Sea for German cruise passengers. In this sense, Rostock and Kiel were the second and third largest German cruise homeports during 2015–2017 [51].

The ports of Helsinki and Stockholm were situated in the ‘Mature Leader’ quadrant during the entire analysed period (see Figs. 6, 7). The ports of Gdansk and Visby dropped from ‘High Potential’ to a ‘Minor Performer’ position in the period 2005–2014. Next, they recovered their initial position of ‘High Potential’ during the period 2015–2019 (see Figs. 6, 7). Both had high fluctuations of its cruise traffic. In the case of Visby port, it registered a huge drop in market share from 4.9% at the beginning to 1.2% at the end of the period analysed. Klaipeda increased its market share during the period analysed, although the net shift was from the ‘High Potential’ position to the ‘Minor Performer’ position (see Fig. 7). The port of Oslo registered the highest loss of market share, dropping to the ‘Minor Performer’ position in the 2015–2019 period. Rønne had an asymmetric evolution. First, it moved from the ‘Minor Performer’ to the ‘High Potential’ position. However, it returned to the ‘Minor Performer’ position during the 2010–2019 period (see Fig. 5).

During the 2000–2014 period, Gothenburg retained the position of ‘High Potential’ and increased its market share. However, during 2015–2019, it dropped to the ‘Minor Performer’ position (see Fig. 7). Riga registered a negative balance during the analysed period. It started as a ‘High Performer’ port due to its high growth rate; however, it dropped to a ‘Minor Performer’ during the remaining subperiods analysed (see Fig. 7). Aarhus moved from the ‘Minor Performer’ position to the ‘High Potential’ position in the three remaining subperiods. The change in the position of Aarhus is related to high growth rates, although its market share increased slightly during the period analysed as well (see Fig. 6).

## Discussion of the results

### Discussion and comparison of the findings with previous research

The level of concentration shown by the Baltic cruise port system, with an average HHI of 0.113 during the period from 2000 to 2019, is similar to the Western Mediterranean port system, which is the leading European cruise destination. According to Pallis and Arapi [9], the Western Mediterranean had an average HHI of 0.066 during the 2005–2014 period. Moreover, Lorenčič et al. [8] obtained similar results for the period from 2010 to 2017. Therefore, both destination regions are unconcentrated. However, the Baltic cruise port system has higher HHI values than the Western Mediterranean. Since 2011, the levels of concentration have declined over time.

The dynamic positioning of the ports in the Boston Consulting Group matrix provides information regarding the changing structures of the cruise port hierarchy within the Baltic Sea. The Baltic cruise port system has a hierarchy ‘picture’ distributed in the four competitive positions of the matrix. Only Stockholm and Helsinki remained during the 4 subperiods analysed in the mature leader position. Copenhagen, St. Petersburg and Tallinn since 2005 moved to become mature leaders. In addition, a relevant change in the hierarchy of ports is represented by the movement of Rostock and Kiel to the best competitive positions since 2005.

If the Baltic Sea is compared with the Mediterranean Sea, some different features can be identified. The first difference is that the number of ports that compose the Mediterranean cruise port network is greater than 70. The vast number of ports in the Mediterranean network also indicates high rates of activity. Comparing the peak season of both destination regions, the Mediterranean Sea registered 22.77 million cruise passenger movements and 9782 cruise calls during the peak season in 2019, whereas the Baltic Sea registered a cruise activity four times lower, with approximately 5.15 million cruise passenger movements and 2653 cruise calls during the peak season in 2019. The second difference is in the size of the ports in terms of throughput; the three largest ports in the Mediterranean registered more than 2.0 million passengers in 2019, whereas the three largest Baltic ports registered less than 1 million passengers.

Furthermore, the hierarchy ‘picture’ of the Baltic cruise port network distributed in the four competitive positions of the matrix is also observed among the 20 major cruise ports of the Mediterranean Sea [9]. Similar results were obtained by Esteve-Perez and Garcia-Sanchez [52] for the Spanish cruise port system, the second country of cruise destination in Europe during 2019. Previous research [9, 52, 53] obtained similar results to those reported for the Baltic cruise port network.

### Theoretical and practical implications of the research

Conceptually, the applied analysis techniques show robustness to determine issues regarding the dynamics of competition in a cruise port network. The application of HHI and dynamic portfolio analysis seems to be a suitable tool combination to assess the competition trends in a given cruise port network. Specifically, applying a dynamic portfolio analysis allows researchers to generate knowledge about how the competitive positions of a set of ports have changed during a specific period. Another advantage of this technique is that it helps to compare the competitive positions of the ports of the network through a graphical overview. The obtained results have a series of practical implications.

The eight dominant ports in the Baltic are located in the best competitive positions, and are ‘Star Performers’ and ‘Mature Leaders’. They have regular and established cruise traffic, and they can be categorised as must-see ports in the Baltic network. These eight ports act as hubs for the distribution of cruise traffic, which benefit the closest ports to them due to the need to create the main element of the cruise traffic, the itinerary. Moreover, the functions of homeport and part-homeport of the leader ports influence the remaining ports since they can attract transit calls. This positive effect can be seen in ports located at high potential, such as Gdansk and Aarhus.

Moreover, the former port has an additional factor in improving its competitive position in the following years, its role of part-homeport. From port operations port of view, mature leader ports register growth rates lower than the average; therefore, it is suggested for these ports to attract calls of ships specialised in luxury and expedition segments to become more diversified ports. In addition, it is recommended to assess new schedules of calls, such as overnight calls and extending the duration of the call far from daytime hours.

Although the cruise season has a different duration, the available number of ports in the Baltic network means that the region could still have strong potential to host more activity. Currently, 14 of 29 ports register fewer than 10,000 passengers per year. These ports have wide possibilities for traffic improvement. In addition, the ports located mainly in the High Potential quadrant and those located in the Minor Performer quadrant could improve their throughput through cooperation strategies with the 8 dominant ports as a win–win mechanism in the process of building cruise itineraries. However, it should be kept in mind the seasonal limitations to develop the activity due to the strong seasonality component of the Baltic Sea, which is mainly associated with icing of the sea. In this regard, the Mediterranean Sea is an annual region with a peak season from May to October [18]. In contrast, the Baltic Sea is a seasonal region with a peak season from May to September and with very low activity, or even without, during the low-season months. Recently, the International Maritime Organisation published a polar code to improve the safety of shipping in ice-covered waters [54]. This code provides an approach for evaluating the risk of vessels when they are navigating in polar areas, helping to determine proper ice-class notation. In this regard, the current advances of the polar shipping industry in new ship designs can be a chance to improve the sailing season in the Baltic Sea during the winter months. In this sense, expedition cruises can be an important niche market for the Baltic Sea because the 2021–2027 order book of cruises for the expedition segment comprises 29 ships [55], some with ice class notation. Therefore, these ships could have the Baltic Sea as an operational area even during winter, overcoming sailing in icing waters.

### Limitations of the research

A limitation of the study is the inability to access the dataset of port origin and port destination of each call registered in the ports analysed. Through these data, it would have been possible to determine the bipartite relations within Baltic cruise ports and the cooperation and competition relationships among them. Moreover, through this information, we could have identified the cruise lines that call at each port. Finally, this would have enabled us to know if there is particular interest in a cruise line because it acts as a port operator in that cruise terminal.

## Conclusions and future perspective of the research

The study provided evidence of the concentration and competitive positions of the Baltic cruise port system since 2000. The Baltic cruise port system has shown strong growth since the beginning of the twenty-first century, which was reflected in a higher number of cruise passenger movements and a higher number of ports hosting cruise activity over time.

The analysis revealed that although the Baltic cruise port system is unconcentrated, eight ports are dominant. The results show a dynamic evolution of competitive positions over time. The portfolio analysis revealed that Copenhagen, Helsinki, Stockholm, St. Petersburg and Tallinn were ports in good dynamic, showing strong competitive positions during the period analysed. The most positive evolutions were associated with the ports of Kiel and Rostock because both moved to become ‘Mature Leaders’, increasing their market shares and registering growth rates higher than the average for the period analysed. In contrast, Oslo dropped from ‘Mature Leader’ to ‘Minor Performer’. Considering the total number of ports in the Baltic Sea, this region will improve its cruise activity and importance among the European cruise destination regions. The formulation of cooperation strategies between the dominant ports and the remaining ports seems to be a key factor to reach higher rates of activity. Two factors appear to be the most important to manage to improve the performance of the whole region: (1) the seasonality of the cruise activity due to weather constraints and (2) the changes that the COVID-19 pandemic will impose on cruise shipping. Advances in polar shipping and the huge orderbook of cruises for the expedition segment could positively affect the Baltic cruise port network, improving the range of activity.

This study helps port managers and stakeholders identify the dynamic performance of a port. In addition, comparisons can be made among competitors taking as reference the results obtained. Moreover, through the obtained results, port managers can plan proper strategies for retaining or improving (as appropriate) their competitive position. These strategies aim to improve the presence of the port in itineraries and/or to build up new cruise itineraries in this region.

Finally, avenues of future research based on the results from this study could be as follows. (1) the study of the dominant cruise market segments that sail in the Baltic; (2) to assess the maximum capacity of current cruise facilities to forecast future developments of port infrastructure; (3) to inventory the type of propulsion systems and power plants of the vessels cruising the Baltic Sea to calculate the carbon footprint of cruise shipping; (4) to measure the extension of the tourist hinterland of each Baltic cruise port; and (5) to assess the sustainability of the cruise activity in the Baltic Sea from economic, social, environmental, and ecological perspectives.

## Data Availability

The datasets used during the current study are available from the corresponding author on reasonable request.
